# A Spinal Arteriovenous Fistula in a 3-Year Old Boy

**DOI:** 10.1155/2014/696703

**Published:** 2014-02-12

**Authors:** Thomas E. M. Crijnen, Sandra van Gijlswijk, Jozef De Dooy, Maurits H. J. Voormolen, Dominique Robert, Philippe G. Jorens, Jose Ramet

**Affiliations:** ^1^Department of Paediatrics, Antwerp University Hospital (UZA), Wilrijkstraat 10, 2650 Edegem, Belgium; ^2^Department of (Paediatric) Intensive Care, Antwerp University Hospital (UZA), Wilrijkstraat 10, 2650 Edegem, Belgium; ^3^Department of Radiology, Antwerp University Hospital (UZA), Wilrijkstraat 10, 2650 Edegem, Belgium

## Abstract

We present a case of a 3-year-old boy with neurodegeneration. Family history reveals Rendu-Osler-Weber disease. Magnetic resonance imaging (MRI) of the spinal cord and spinal angiography showed a spinal arteriovenous fistula with venous aneurysm, causing compression of the lumbar spinal cord. Embolisation of the fistula was executed, resulting in clinical improvement. A week after discharge he was readmitted with neurologic regression. A second MRI scan revealed an intraspinal epidural haematoma and increase in size of the aneurysm with several new arterial feeders leading to it. Coiling of the aneurysm and fistulas was performed. Postoperative, the spinal oedema increased despite corticoids, causing more extensive paraplegia of the lower limbs and a deterioration of his mental state. A laminectomy was performed and the aneurysm was surgically removed. Subsequently, the boy recovered gradually. A new MRI scan after two months showed less oedema and a split, partly affected spinal chord. This case shows the importance of excluding possible arteriovenous malformations in a child presenting with progressive neurodegeneration. In particular when there is a family history for Rendu-Osler-Weber disease, scans should be performed instantly to rule out this possibility. The case also highlights the possibility of good recovery of paraplegia in paediatric Rendu-Osler-Weber patients.

## 1. Introduction

When confronted with a paediatric patient with developmental degeneration, the most likely causes are a (congenital) neurologic or metabolic disorder, a genetic defect or syndrome, exposure to a toxic agent, or an infection of the brain or nerve system [1]. The main localisation of such disorders must be searched for in the cranium.

With this case report we want to emphasize that an arteriovenous malformation (AVM) might also be responsible for the symptoms of regression, especially when there is a suspicion of Rendu-Osler-Weber disease (ROWD), an autosomal dominant vascular disorder with a variety of clinical manifestations. In this disease epistaxis, gastrointestinal bleeding, and iron deficiency anaemia are most commonly reported, along with characteristic mucocutaneous telangiectases [2]. In addition, AVMs might occur in the pulmonary or hepatic circulations or cerebrally as well as in or around the spinal cord as arteriovenous fistulas (AVFs). These complications demand knowledge of the risks and benefits of screening and treatment in affected patients [3, 4].

We report on a 3-year-old boy, initially presenting with developmental regression. A spinal AVF was diagnosed requiring embolisation and appropriate treatment. We highlight two unusual features: the observation that a spinal AVF might also be responsible for symptoms of regression and the apparently good recovery after acute paraplegia.

## 2. Case

A 3-year-old boy was brought into hospital because of developmental regression. His medical history revealed little problems, except for a bronchiolitis caused by respiratory syncytial virus, for which he had been in hospital as a toddler. Uptill a few months earlier his neuromotor development had always been on schedule. Six months before admission to our hospital he was hospitalized in a different clinic with convulsions, thought to be caused by a viral infection with enterovirus. Afterwards he progressively developed constipation, limping, and an inability to play in his normal manner. The boy is the firstborn son of two nonconsanguineous parents of Afro-Caribbean origin. The mother and maternal grandmother of the boy are known to have ROWD. A maternal uncle was diagnosed with systemic lupus erythematosus.

**Figure 1:**
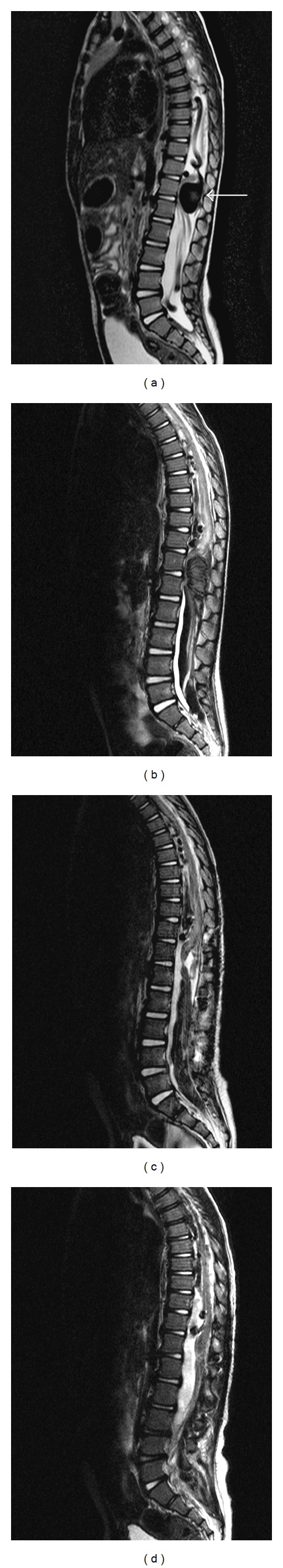
Thoracolumbar MRI scans, midsagittal views, and T2 weighted images: (a) scan at presentation of the child shows the dilated vascular intraspinal structures and the large aneurysm (arrow) nearly completely involving the spinal canal at the level of T12 with spinal cord compression; (b) scan after second embolisation shows the haematoma (dark) in the lower spinal canal, a decrease in size of the dilated intraspinal blood vessels, the occluded large aneurysm and the spinal cord oedema/hyperintense lesions of the lower spinal cord; (c) scan after surgery shows the resected aneurysm, with a very thin and split spinal cord at level T12, residual intraspinal haematoma, some dilated thrombosed intraspinal vessels, and spinal cord oedema/hyperintense lesions of the lower spinal cord; (d) scan two months after treatments shows a resolving of the haematoma, unchanged thrombosed intraspinal vessels, and a decrease in oedema/hyperintensity of the lower spinal cord.

**Figure 2:**
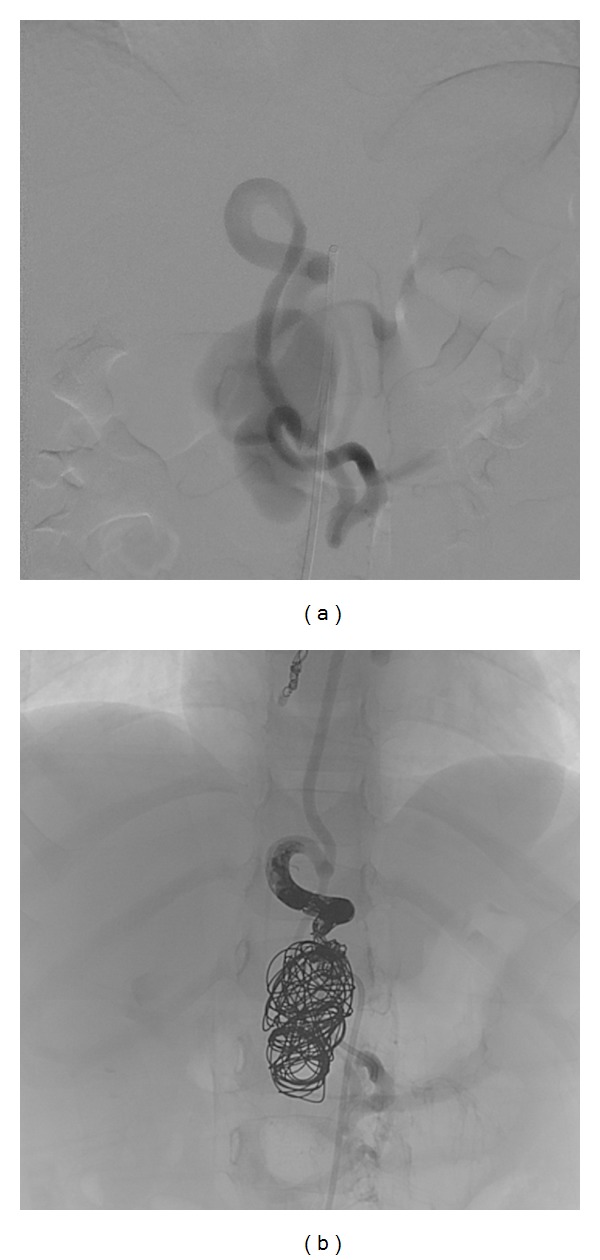
Angiographic imaging: (a) spinal angiogram, arterial phase, anteroposterior view, shows the arteriovenous fistula and the giant aneurysm at the level of T12/L1; (b) X-ray, AP view, same level, shows the coils and Onyx cast after endovascular occlusion.

During the diagnostic workup in a neighbouring general hospital an MRI scan of the spinal cord was performed (Figure 1(a)). It showed a spinal vascular malformation with a large aneurysm at the level of the lower thoracic spinal cord, causing massive compression of the lumbar spinal cord. The boy was transferred to our university hospital for further diagnosis. Spinal angiography (under general anaesthesia) identified the vascular malformation as a spinal arteriovenous fistula with an associated large venous aneurysm at the level of the fistula. The dilated arterial feeder as recognized from MRI originated from the intercostal artery at T9. It was thought to be a single fistula. Due to the low weight of the boy, only a limited amount of contrast could be administered and more distal thoracic levels were not checked. An embolisation of this AVF at T9 was carried out, using a coil and a liquid embolic agent (Figure 2). The idea was that the aneurysm would gradually shrink in time, whereas initial aneurysm occlusion would result in an oedematous reaction with increase in spinal cord compression. Postoperative high-dose dexamethasone was given to prevent oedema of the spinal cord. The boy recovered well with spontaneous movements of the lower limbs and preservation of sensibility. Because of his symptoms and the family history, genetic testing was started up to try and confirm the diagnosis of ROWD in the boy.

A week after discharge he was readmitted with severe pain in his back, stiffness of the neck, and an inability to walk. A complication of the embolisation was suspected. A second MRI scan revealed an intraspinal epidural haematomata expanding caudally from level L1, at the site of the known venous aneurysm that had also increased in size (Figure 1(b)). A new spinal angiography, because of the acute state now covering more thoracolumbar levels, demonstrated several other arterial feeders of the AVF. During the same session, coiling of the aneurysm and the fistulas was performed. Afterwards the paraplegia of the lower limbs was more significant and the mental state of the boy deteriorated. Again, corticosteroids were administered to reduce oedema. However, a new MRI scan showed increased oedema of the spinal medulla (Figure 1(c)). A surgical laminectomy was performed, the haematoma was drained, and the coiled aneurysm was removed to release the spinal compression. Due to the long-standing compression the spinal cord was split and very thin at the level of the aneurysm.

After the operation the boy recovered remarkably well. He stayed in our hospital for a month. During that period of time his mental state completely normalised and he was able to start walking again. He did however develop a hypertonic bladder, for which he has to be catheterised. An MRI scan performed two months later showed decrease of oedema of the spinal medulla (Figure 1(d)). Genetic testing did confirm the diagnosis of ROWD. Further rehabilitation is tended to at home by a paediatric physiotherapist.

## 3. Discussion

Rendu-Osler-Weber disease is an autosomal dominant hereditary condition, caused by a mutation in the gene encoding endoglin (ENG), a transforming growth factor-beta binding protein that has an angiogenic function, on chromosome 9q34.11 [5–7]. The disease is also known by the name “hereditary haemorrhagic telangiectasia (HTT) type 1.” The incidence of HTT is estimated to be 1/5000 to 8000 newborns [8]. Patients display a vascular dysplasia, as a result of which they develop haemorrhagic telangiectases and AVMs of mucosa, viscera, and skin. Complications of mucosal dysplasia are epistaxis and gastrointestinal tract bleedings [9]. The Curaçao criteria have been defined to improve clinical diagnosis and management [2]. HHT is to be considered if some of the following criteria are present: (1) spontaneous recurrent epistaxis; (2) multiple telangiectases at characteristic sites (nose, lips, oral cavity, and fingers); (3) family history (a first-degree relative with HHT); and (4) visceral lesions (gastrointestinal telangiectases with or without bleeding, or pulmonary, hepatic, cerebral or spinal AVMs). If three or more criteria are present the diagnosis is definite. A patient with two criteria is considered a possible case. Otherwise HHT is unlikely. Our patient presented two criteria and was therefore a likely patient. Genetic testing, however, could confirm our suspicion.

The mean age of onset for the epistaxis in ROWD is 12 years. By the age of 21, over 90% of all mutation carriers have developed epistaxis [10]. Bleeding may be excessive and can occur regularly. Cauterization and sometimes even surgery are needed in such cases [9]. The mucosa of the tongue, oral cavity, and lips and the fingers are also frequently affected, showing distinct telangiectasic lesions. In 74% of cases telangiectases are documented, half of them being younger than 30 years of age [3]. Intestinal bleeding usually is mild and no invasive treatment is needed. In case of anaemia a patient can be started on iron supplementation. If severe bleeding tends to be present, endoscopic argon plasma coagulation or arterial embolisation can be performed to stop the haemorrhage [11]. Skin lesions in the form of telangiectases occur mainly in the face and on the fingers, with a frequency of about 33% and 41%, respectively. Normally, no treatment is needed. In case of disfiguring lesions in the face treatment with laser may be effective [3].

Patients can develop AVMs in several visceral organs, most commonly in lungs, liver, and brain [3, 4]. AVFs in the spinal cord are very rare (<1%), but children more frequently present with an AVF in the spinal cord as a first sign of the disease [12]. Coiling of AVFs is often necessary to prevent complications of haemorrhage or compression of other structures. In our case, the AVF primarily caused compression of the spinal medulla, which led to the developmental regression. Haematoma and oedema, arising after the treatment, led to secondary neurological deficit. The consequences of these symptoms are severe and should be treated immediately. Compression of the spinal cord might cause irreversible damage if it exists for a long period of time.

## 4. Conclusion

The possibility of a spinal AVF in children presenting with progressive developmental regression should be considered. Especially in case of a family history for Rendu-Osler-Weber disease, scans should be performed instantly to rule out this possibility. Invasive treatment must be performed as soon as possible to minimise damage to the spinal cord. Close observation and follow-up afterwards are crucial in the prevention of secondary injury.
